# Some Cases of Drug Habit*Read before the Atlanta Society of Medicine, February 3, 1898.

**Published:** 1898-04

**Authors:** C. C. Stockard

**Affiliations:** Atlanta, Ga.


### SOME CASES OF DRUG HABIT.*

By C. C. STOCKARD, M.D.,

ATLANTA, GA.

I feel that I ought to apologize to those present for presenting a subject in which so few of us, apparently, are interested. It is because of the fact that I think the profession needs arousing to the importance of drug habituation, for the benefit of society in general, and also that such a large number among our own ranks

*Read before the Atlanta Society of Medicine, February 3, 1898.

are falling victims to morphia, cocain, etc., that I call your attention to the steady increase in the number of drug habitués, and the fearful consequences to all connected with the victims. Since the invention of the hypodermatic syringe these cases have rapidly increased, as shown by the numerous advertised cures and institutions for the treatment of them, in both the lay and medical press.

There seems to be a fascination about the syringe which causes the patient to more rapidly increase the doses taken than when used by the stomach, and the evil consequences are proportionately greater. I have on hand now a patient who, after twenty years as an opium habitué, came to me taking one drachm of laudanum three times a day. On the other hand, in a recent letter from a lady in regard to her brother she stated that he had used morphia hypodermatically twelve years and cocain four, and that last year he had run the cocain up to three drachms per day, and she didn't know how much morphia.

I heard recently of a morphinist, himself treating the habit, who took within forty-eight hours ten drachms. It is only a matter of wonder that this sort of drugging does not kill more rapidly than it does.

The treatment of drug habitués fails of success in a large proportion of cases because few of them are able to pay for treatment sufficiently long to insure permanent results. The customary method is to send the patient home—or, rather, for him to go home for want of money—after remaining four to eight weeks in an institution, and the too frequent consequence is that, owing to sleeplessness or some slight ailment, he returns to his syringe, when a few months more of building-up treatment and proper moral support would have been sufficient to enable him to resist in the future.

It seems that the time is not far off when it will be necessary for legislatures to make provision for the care of these people, as is done for the insane; and also more rigid laws governing the sale of drugs likely to be bought by habitués.

I give below brief histories of a few cases I have treated in the past two years:

CASE 1.—Dr. ——, aged about fifty, a man of fine physique, highly educated and intelligent; had been a successful specialist in a Northern city. He had been using morphia hypodermatically for twenty years, having contracted the habit while resident physician of a Boston hospital. He stated that he had been broken of the habit once or twice. He was taking twenty grains per day in four or five doses. Had at one time taken cocain; had been a heavy drinker of beer and whisky, and frequently put himself to sleep inhaling chloroform. I followed with him the Mattison plan, taking away the morphia in about ten days; but at the end of five weeks, at which time he left, he was still taking two or three grains of codeine phosphate per day, and though he repeatedly told me that he left off the codeine and took no morphia until about four months after, when he had an inflammation of the knee which has laid him up the past eighteen months, still I always believed that he went back to his morphia immediately after leaving me. During and after the withdrawal of the drug he suffered from cramps, vomiting, diarrhea and sleeplessness. He was continually threatening to kill himself and made several attempts—one time removing the tube from a small gas stove, turning on the gas, and going to bed with the tube in his mouth.

CASE 2.—Dr. ———, four years before coming to me, had begun the use of morphia while suffering from an infected finger. He had at times combined cocain with morphia, and when he came to me had been using cocain for a week or two. This was immediately withdrawn, and the morphia rapidly, so that in four weeks he was in very good condition and insisted on returning to work. About two months later he returned, stating that for a cold he had taken antikamnia and codeine, and the next morning a hypodermic of morphia. This occurred about three weeks after leaving me, and on his return he was using about three grains per day. At this time (just a short while before moving into the Atlanta Retreat) I was not prepared to care for such a case properly, but, as he had gotten on so nicely before, I anticipated no trouble and undertook the case. Things went very well till his dose of morphia was getting pretty small, when, one night, though I had taken the precaution to take his shoes from the room, he went out shod with slippers, filled up on whisky, and also supplied himself with cocain. After a few days he began to write voluminous letters and poetry, and then a few days later hallucinations developed. He thought the people in the house were watching his actions and were talking about and planning against him. These hallucinations grew steadily worse until the sparrows singing in the streets were talking about him, the ticking clock was a telegraph machine of some sort, through which people were communicating about and plotting against him. After his worst day, and when I feared I should have to send him to Milledgeville, I gave him at bedtime fifty grains of sodium bromide and thirty of trional. He went to sleep promptly and slept soundly until half-past five next morning, when he sprang from his bed yelling, "What does this mean?" I went in and spoke to him, when he said, "Thank God! it isn't done, but you didn't keep it from being done." I asked, "What are you talking about?" when he replied, "Those men were trying to castrate me by electricity." I had the night previous arranged it, and immediately telephoned for a policeman, who took him to the police station to keep him until I could arrange for sending him to the asylum. Here he soon began to sober up, and told me that he had been taking cocain. He got no more, and in forty-eight hours had sufficiently recovered his reason for me to take him back home. At evening, for a few days, there would be some return of hallucinations, but he improved steadily and in a few weeks returned to his home, and is now doing well at practice, having gone about a year without relapse.

CASE 3.—Dr. ——. This case I relate to illustrate how one who is not of the temperament, or has not inherited neurotic tendencies predisposing to this sort of thing, can take morphia a long time without carrying it to the excess common to habitués. For six years this man has suffered from myelitis, produced by accident, being completely paralyzed from the waist down, with inability to void feces or urine, vomiting during nearly all these years from one to fifty times a day, and feeling all the time as though a log were lying across his body, crushing it; and yet was taking only two and a half grains of morphia per day. In this condition he does considerable practice, performing a good many surgical operations. A man of remarkable courage and nerve he is. He thought his general health had improved latterly, and that if he could leave off his morphia he would be better, but after a few weeks' trial I advised him not to go further with it, believing that there was nothing else which would give him any relief from his pain with as little injurious effect.

CASE 4.—Mrs. ——, aged about thirty, had used morphia hypodermatically for five years, and was taking twenty-five grains per day. Had twice been treated by a guarantee cure establishment, but was given up because she had an inflamed external hemorrhoid, which the man in charge, not a physician, did not know how to relieve, and hence advised her that she could not stand it without morphia. I had less trouble with her than with any case I have ever treated, getting her entirely off in four weeks. Though her general health is not good, up to a few weeks ago she was still holding out, though, living in the house, as she does, with two habitués, it will be remarkable if she does not go back to it.

CASE 5.—Dr. ——, aged about forty-five, was struck by lightning about two years ago, and afterward had typhoid fever. These were followed by the development of attacks resembling angina pectoris. He would be taken suddenly with violent pain in the cardiac region, which he described as feeling like a red-hot knitting needle was thrust through the heart and withdrawn. With these attacks were spasms of various muscles, half-purposive movements, and I promptly told him that he had a good case of hysteria. Drs. Pinckney and Hagan both saw him and agreed with my diagnosis, the latter suggesting a trial of hypnotism, for which he proved to be an unusually ready subject. After Dr. Hagan had hypnotized him I repeated it a number of times. While hypnotized he was given the suggestion that he would have no more of the spells and would want no morphia. The first day he had no morphia he complained some of his knees, and said that if I would brace them he would be all right. They were braced by hypnotic suggestion, and that was the last of it. There was never a spell after the first hypnotism, and now, after three months, he is perfectly well—no spells, no desire for morphia. He has been gaining about half a pound a day.

I believe with Dr. Howard, of Baltimore, that in proper subjects hypnotism will give better results in drug habits than any other method or treatment.

CASE 6.—Dr. ———, aged thirty-one, a man with no family history of neurotic predisposition, about four years ago had his druggist give him a snuff-powder, for cold, which he was in the habit of prescribing. From this start he had continued and soon used the pure hydrochlorate of cocain, always snuffing it up the nostrils. Two months before coming to me he had left off the cocain and took, for nervousness and sleeplessness, bromodine, and was taking at the time of coming to me two ounces per day. So he was taking half an ounce each of chloral and potassium bromide, besides cannabis indica, hyosciamus, etc. I put him on tonic treatment, gave trional for a few nights, and left off bromide at once. He improved rapidly and went home at the end of three weeks. In a letter recently received he says he is well. Not being of neurotic disposition, I think he got into this habit purely by accident, and I do not apprehend any relapse in his case.

It will be seen that five out of these six cases reported are doctors. This does not represent the proportion of doctors among all the cases I have treated; still, the proportion is very large—sixty-six per cent. This large proportion of doctors is accounted for by the fact that doctors are less disposed to go to the advertising quacks, but still there are a larger proportion of doctors addicted to drug habits than any other class of our citizens, and I would particularly warn my professional brothers to be cautious about taking any of these drugs, and especially by the hypodermatic method.

---

### CODEINE AS ANALGESIC.

The following solution of codeine is recommended by Dr. I. J. Jones, of Austin, Texas, as a good analgesic mixture in place of morphine.—(*Texas Med. News.*)

| | | |
|---|---|---|
| ℞ | Codeine sulph .................................... | gr. xxxii. |
| | Spir. amm. ar.................................... | ʒ vi. |
| | Sp. rect .................................... | ℥ i. |
| | Syr. aurant. cort .................................... | q. s. ℥ iv. |

Sig.: One to three teaspoonfuls once or twice daily.

